# Comparison of the Tuberculin Skin Test and Interferon Gamma Release Assay for the Screening of Tuberculosis in Adolescents in Close Contact with Tuberculosis TB Patients

**DOI:** 10.1371/journal.pone.0100267

**Published:** 2014-07-14

**Authors:** Seung-Eun Song, JiYeon Yang, Kil Soo Lee, Hyungjun Kim, Young Mi Kim, Seonghan Kim, Mi-Sun Park, Su Yeon Oh, Jin Bum Lee, EunPyo Lee, Sang-Hee Park, Hee-Jin Kim

**Affiliations:** 1 Division of Tuberculosis and Bacterial Respiratory Infections, Korea National Institute of Health, Cheongwon-gun, Chungcheongbuk-Do, Republic of Korea; 2 The Korean Institute of Tuberculosis, Cheongwon-gun, Chungcheongbuk-Do, Republic of Korea; The Catholic University of the Sacred Heart, Rome, Italy

## Abstract

**Background:**

The tuberculin skin test (TST) frequently yields false positive results among BCG-vaccinated persons thereby limiting its diagnostic value particularly in settings with high BCG vaccination rate. We determined the agreement between IGRA and TST using 2 cutoff values and identified possible relationships between the results of these tests and the development of active tuberculosis.

**Methodology:**

Adolescents aged 11–19 years in close contact with smear-positive tuberculosis cases and with normal chest radiographs were recruited from middle and high schools in South Korea. The TST was conducted by trained nurses, and blood was drawn for the QuantiFERON-TB Gold In-Tube (QFT-GIT). Participants were followed up for 2 years to check for incidence tuberculosis.

**Results:**

A total of 2,982 subjects were included in the study, the average age was 15.1 years (SD 1.3), 61% had BCG vaccination scars. The agreement of QFT-GIT and the TST was low (κ = 0.38, 95% CI 0.32 to 0.42) using 10 mm cutoff; however, when the 15 mm cutoff was used, the agreement was intermediate (κ = 0.56, 95% CI 0.50 to 0.61). The odds ratio (OR) for the development of active tuberculosis was 7.9 (95% CI 3.46 to 18.06) for QFT-GIT positive patients, 7.96 (95% CI 3.14-20.22) for TST/QFT-GIT+ and the OR 4.62 (95% CI 2.02 to 10.58) and 16.35 (95% CI 7.09 to 37.71) for TST 10 mm and 15 mm cutoff respectively.

**Conclusions:**

The results of this study suggest that the TST cutoff point for patients aged 11–17 years would be 15 mm in other study. The OR of QFT-GIT for the development of active tuberculosis and its intermediate agreement with TST using 15 mm cutoff demonstrates its role as an adjunct diagnostic tool to current clinical practice. Positive responders to both TST and QFT-GIT at the outset may benefit from chemoprophylaxis.

## Introduction

For about a hundred years, the tuberculin skin test (TST) has been a diagnostic tool for determining previous infection with *Mycobacterium tuberculosis (M. tb)*. This test lies on the principle of detecting a delayed hypersensitivity reaction to tuberculin antigens, of which the purified protein derivative (PPD) is the most important. PPD is a cell-free purified protein fraction obtained from a human strain of *M. tb* and consists of more than 200 proteins. This reaction produced by the TST may occur in patients with active tuberculosis, latent tuberculosis infection (LTBI) or in those previously immunized with the BCG vaccine. The delayed hypersensitivity reaction, however, is non-specific for all *Mycobacterial* infections. Thus, TST has a lower specificity in populations with high BCG coverage and NTM exposure such as South Korea [1]. False positive tuberculin reactions occur in individuals who have been infected with other *Mycobacteria* because of some antigens shared within the genus [2].

Recently, the interferon gamma release assay (IGRA), a T-cell based assay using *M. tb-*specific antigens, has been increasingly used and studied for the diagnosis of both active or latent tuberculosis (TB) infections. This test relies on the production of interferon gamma, a potent proinflammatory cytokine released by T-cells and NK cells and is reflective of adaptive T-cell responses to TB. The IGRA, used together with other tests, has been introduced into clinical practice to assist in the diagnosis of active tuberculosis in many countries [3]. In 2005, the earliest version of the test (QuantiFERON-tuberculosis Gold test or QFT-G, Cellestis Limited, Carnegie, Victoria, Australia) was approved by the US Food and Drug Administration together with the publication and release of the CDC guidelines for its use [4], [5]. The QFT-G was the first blood test approved for the diagnosis of LTBI [4]. According to the CDC guidelines, QFT-G can be used in all circumstances for which the TST is currently being used, including contact investigations, evaluation of recent immigrants who have been vaccinated with BCG, and tuberculosis screening of health-care workers and others undergoing serial investigation for *M. tb* infection [5].

In theory, the IGRA offers a more accurate approach than the TST for the identification of individuals who have LTBI and might improve tuberculosis control by allowing for more precise targeting of preventive treatment [6]. According to published studies, the IGRA is a better indicator of the risk of *M. tb* infection than the TST especially among BCG-vaccinated children [7], [8]. Other studies report that the use of a confirmatory IGRA for TST contacts could effectively focus targeting of LTBI treatment to fewer contacts in a TB-intermediate-incidence setting with a high BCG-vaccination rate such as South Korea [9].

More information is needed to determine whether the TST or the IGRA can more accurately diagnose tuberculosis. Determination of a “gold standard” diagnostic test for the diagnosis of LTBI requires evidence-based evaluation of these two candidate assays, TST and the IGRA, in various population groups. In this article, we focus on the strengths and limitations of TST and QuantiFERON-TB gold In –Tube (QFT-GIT) that that are available for the diagnosis of LTBI in middle and high school students in close contact with tuberculosis patients in South Korea. We determined the agreement between IGRA and TST using 2 cutoff values and identified possible relationships between the results of these tests and the development of active tuberculosis.

## Materials and Methods

### Study Design

We performed a prospective cohort study among close contacts of smear-positive tuberculosis patients.

### Participants

Between 2008 and 2012, the Korean Institute of Tuberculosis performed screening for tuberculosis among close contacts aged 11–19 years of identified smear-positive tuberculosis cases in 45 middle and high schools in South Korea. Chest radiographs were performed and patients with abnormal chest x-ray findings were given anti-tuberculosis chemoprophylaxis and were excluded from the study. Only contacts with normal chest X-ray were included in the study for reducing selection bias. Participants were excluded when: (1) they showed abnormal findings in simple chest radiographs, (2) they had taken immunosuppressive agents or anticancer drugs earlier, and (3) they had been treated with antituberculous drugs or chemoprophylaxis earlier.

Written informed consent forms were obtained from all participants prior to the enrollment in the study. Research nurses interviewed the participants and gathered important demographic and clinical data such as place of birth, history of tuberculosis exposure outside the school, previous and current chest x-ray results and other pertinent medical history. Physical examination was likewise performed including visual inspection for the presence of BCG scars. TST was performed after blood collection for the QFT-GIT.

### Definition of Terms

Close contacts of smear-positive tuberculosis index cases can be their classmates, exchanging classmates or club mates. A classmate was defined as a student who spent most time with the index case in the same classroom. An exchanging classmate was defined as a student who was in contact with the index case in other classrooms for more than 1 hour per week. A member of club mates are was defined as a student who was in contact with the index case only during extracurricular activities.

### TST

The TST was administered by intradermal injection (0.1 ml) of 2 tuberculin units of purified protein derivative (RT 23; Statens Serum Institute, Copenhagen, Denmark) into the anterior surface of the forearm with a disposable syringe and a 27-gauge needle by using the Mantoux technique. The maximal transverse size of induration was read 48–72 hours later with a ruler or a caliper by a research nurse.

### IFN-γ Release Assay (QFT-GIT)

QuantiFERON tuberculosis Gold In-Tube [QFT-GIT] (Cellestis Inc, Valencia, CA) tests were performed according to the manufacturer's instructions. Briefly, whole blood was collected by venipuncture from each subject at the date of injection of PPD and incubated for 16–24 hours in 3 separate conditions: 1) a mixture of 3 TB antigens from RD1 and RD11 (ESAT-6, CFP-10, and TB7.7); 2) a mitogen as a positive control; and 3) a mock stimulation as a negative control (nil). Following the stimulations, 150 µL of the supernatant was harvested from each tube. Then, 50 µL of each supernatant was used to determine its interferon gamma (IFN-γ) concentration by the ELISA. A QuantiFERON value of 0.35 international units or more was deemed positive according to manufacturer's instructions. To eliminate the possibility of false-positive IGRA results due to PPD reagents, blood samples were collected before PPD injection.

### Data Analysis

Data obtained from the school surveys were coded on a survey instrument and edited by the field interviewers. Field and laboratory data were computerized using specially programmed entry software.

Agreement between the TST and QFT-GIT results was analyzed using Cohen's Kappa coefficient (*k*), with the following criteria being applied in the interpretation of values: *k*≤0.20 (poor agreement); 0.21≤*k*≤0.40 (weak agreement); 0.41≤*k*≤0.60 (moderate agreement); 0.61≤*k*≤0.80 (good agreement); *k*≥0.81 (very good agreement) [10].

Univariate analysis was performed to examine the distribution of the initial TST and QFT-GIT results. The strength of associations between the results and development of tuberculosis was assessed using Fisher's exact test.

We assessed crude associations between the covariates and the outcomes (TST ≥15 mm and QFT-GIT ≥0.35 IU/ml) by computing odds ratios and corresponding 95% confidence intervals. Multivariate logistic regression was used to estimate associations between the covariates and the outcomes, adjusting for potential confounders that showed a significant effect in crude odds ratios at the 5% significance level.

Data management and statistical analyses were performed using IBM SPSS Statistics 19 (SPSS Inc., Chicago, IL). The individuals in this manuscript and their parents have given written informed consent to publish these case details. The study was approved by the research ethics committee (Korean Institute of Tuberculosis institutional Review Board KIT-2008-03).

## Results

**Figure 1:**
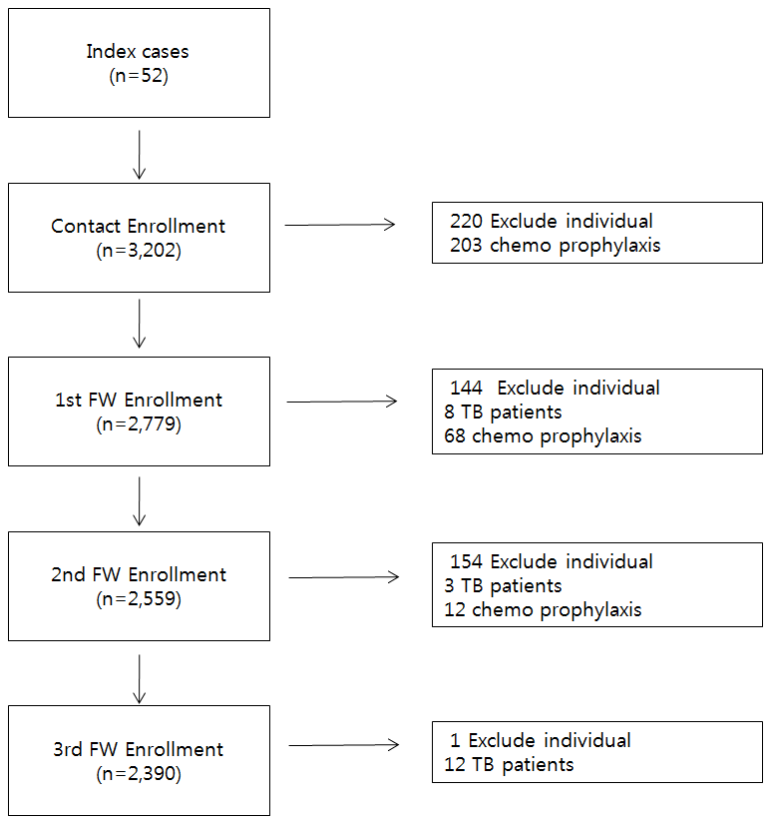
Study profile for recruited subjects by setting. Index case  =  the initial patient in the population of our epidemiological investigation; Exclude individual  =  disagree to this study, lost to follow-up; TB  =  tuberculosis; chemoprophylaxis  =  received recommendations for use of an isoniazid to treat latent *Mycobacterium tuberculosis* infection.

A total of 3,202 close contacts with normal radiographic findings from 8 middle schools and 37 high schools were identified and invited to participate in the study (Figure 1). Blood samples were taken from the participants immediately before the present follow-up study, and 6, 12, and 24 months after the present follow-up study. Participants were excluded when: (1) they had received chemoprophylaxis earlier, (2) they had received immunosuppressive chemotherapy or anticancer therapy earlier, and (3) active/inactive tuberculosis was found in radiographs. Of these, 220 did not give consent to participate in the study. Table 1 shows baseline demographic and clinical characteristics of the 2,982 subjects who were included in the analysis. The average age was 15.1 years (SD 1.3), 54.5% were male. Twenty participants (0.7%) reported a previous family history of tuberculosis. BCG vaccination scars were observed in 61.0% (n = 1,818) of participants.

**Table 1:** General characteristics of the study population.

		Total	
		N	%
Total		2,982	100.0
Age (mean ±SD)		15.1±1.3	
Region	Seoul	889	29.8
	Gyeong-gi	1,126	37.8
	Incheon	474	15.9
	Other	493	16.5
BCG scar present	Yes	1,818	61.0
	No	1,164	39.0
Family history of TB	Yes	20	0.7
	No	2,962	99.3
Relationship with index case	Classmates*	1,499	50.3
	Exchanging classmate†	907	30.4
	Club mates‡	82	2.7
	Others	494	16.6
Smoking	Non-smoker	2,348	78.7
	Current-smoker	157	5.3
	Ex-smoker	123	4.1
	No response	30	1.0
	Not follow up	324	10.9
BMI§	mean ± SD	21.0±3.4	
	median(IQR)	20.3(18.8, 22.6)	
	<18.5	633	21.2
	18.5–25.0	1,997	67.0
	>25.0	352	11.8

^*^Classmates: The same classmates with index case.

† Exchanging classmate: who contact with the index case in other classroom over 1 h per week.

‡ Club mates: contact with the index case during extracurricular activities.

§ BMI: body mass index.

At the end of the 2 year follow-up period, a total of 23 out of 2,982 patients (0.8%) developed and were treated for active tuberculosis. Eight subjects were diagnosed within first follow up period, 3 subjects were diagnosed for second follow up, 12 subjects were diagnosed after second follow up. The average time for development of active tuberculosis was 14.4 month after study enrollment. Twenty-one out the 23 patients who developed active TB were positive for both TST and QFT-GIT.

**Figure 2:**
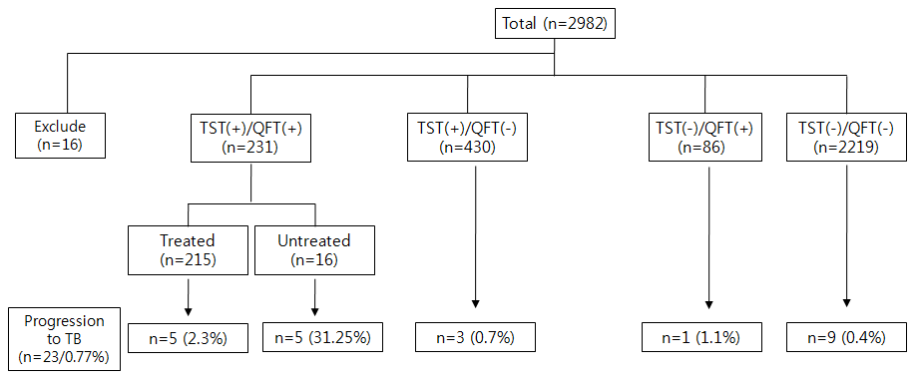
The classification and yield of progression to TB our study subjects. TST (-)  =  tuberculin skin test-negative; TST (+)  =  tuberculin skin test-positive; QFT-G (-)  =  QuantiFERON-TB gold assay-negative; QFT-G (+)  =  QuantiFERON-TB gold assay-positive; Treated  =  isoniazid (INH) was used for chemoprophylaxis.

As shown in Fig. 2, 16 participants were excluded from the study because their IGRA results were indeterminate for TB-Antigen responsiveness; it cannot include the classification and progression to TB in our study.

The participants were classified into 5 groups according to TST, QFT-G results, and LTBI treatment: (1) TST +/QFT-G +, treated (5 of 215, 2.3%); (2) TST +/QFT-G +, untreated (5 of 16, 31.25%); (3) TST +/QFT-G - (3 of 430, 0.75%);(4) TST -/QFT-G + (1 of 86, 1.1%); and (5) TST -/QFT-G - (9 of 2219, 0.4%). TST and IGRA positive results were found with development of tuberculosis, and 3 participants receiving preventive treatment failed to achieve optimal care.

TST positive test results based on induration size cutoff at 10 mm and 15 mm are shown in Table 2. When the 10-mm induration cutoff was used, 2,319 subjects (77.8%) had negative TST results, and 663 subjects (22.2%) had positive TST results. When the 15 mm cutoff was used, 2,751 subjects (92.3%) had negative TST results, and only 231 subjects (7.7%) had positive TST results. The difference in the proportion of patients with positive results between the 2 cut-offs were statistically significant (z = 16.081, p = 0).

**Table 2:** Agreement between the results of the tuberculin skin test and the QFT-GIT test.

		QFT				
TST		Positive	Negative	Indeterminate	Total	Kappa (95%CI)
TST (≥10 mm)	Positive	231	430	2	663	0.383
	Negative	86	2,219	14	2,319	(0.342, 0.424)
TST (≥15 mm)	Positive	163	68	0	231	0.555
	Negative	154	2,581	16	2,751	(0.503, 0.607)
Total		317	2,649	16	2,982	

For the QFT-GIT, 317 (10.65%) of the 2,982 subjects had positive results. The agreement of QFT-GIT and the TST using 10 mm cutoff was low (*κ* = 0.383, 95% CI 0.324 to 0.424). When the TST using 15 mm cutoff was used, the agreement between QFT-GIT and TST was intermediate (*κ* = 0.56, 95% CI 0.50 to 0.61).

At the end of the 2-year follow-up period, the percentage of those who developed active tuberculosis was 1.96% (13 out of 663) in TST-positive subjects with a cutoff induration of 10 mm, while it was 3.47% (11 out of 317) in QFT-GIT-positive subjects. The odds ratio for the development of active tuberculosis was 7.9 (95% CI 3.46 to 18.06) for QFT-GIT while for TST, the OR is 4.62 (95% CI 2.02 to 10.58) and 16.35 (95% CI 7.09 to 37.71) for 10 mm and 15 mm cutoff respectively.

Multivariate analyses adjusted for age, sex and BCG scar for risk factors associated with a positive TST (for both 10 mm and 15 mm cutoff) and QFT-GIT was performed. However, there were no statistically significant factors identified (e.g. type of close contact with the index case).

## Discussion

Describing the relationship between contact history and sporadic outbreaks or transmission of tuberculosis is important for tuberculosis control especially in settings where the burden of tuberculosis is considered intermediate or low. In such cases, two strategies play vital roles in the eventual success of tuberculosis control: (1) treatment for LTBI to prevent subsequent reactivation and transmission and (2) improved accurate diagnosis and identification of peoples with high risk of tuberculosis, including improved diagnostic capabilities for LTBI, as in cases of outbreak or community transmission. The experience with TST has been more extensive and its cost significantly cheaper; however, it has a low specificity and a high false positive rate in countries with high coverage of BCG vaccination. On the other hand, IGRA is costly, but it has a high specificity. The finding in this study that TST at 15 mm cutoff had high OR for the development of active tuberculosis shows that TST (15 mm cutoff) still has a role in clinical practice. In Korea, similar with the UK, 2-step or hybrid methods in addition to the IGRA are carried out to detect LTBI in the TST-positive subjects [11]. The Korean guidelines for national tuberculosis control recommend IGRA tests even if the TST result is positive.

In this study, 23 out of 2,982 (0.77%) close contacts developed active tuberculosis. This figure is similar with a recent study that reported the incidence of tuberculosis in close contacts to be as high as 0.99% [12]. The lower incidence in our case, however, is because the 231 subjects who were positive on both tests were treated for latent tuberculosis infection with preventive chemotherapy. In addition, we chose to investigate a moderate-risk population according to the national TB control guidelines instead of other subjects at high risk of transmission.

Another limitation of this study was that we were not able to confirm that the *M. tb* strain of those who developed active tuberculosis was of the same strain as the index case. Because they have bacteria culture negative result.

In our study, initial TST-positive rates were higher than IGRA-positive rates (22.2% and 10.6% at a TST cutoff of 10 mm). Considering that 61% of the patients included in the study had received BCG vaccine, this difference can be attributed to earlier reports that TST results are affected by prior exposure to *Mycobacterial* species such as BCG vaccination, (causing false positive results). However, a meta-analysis conducted by Farhat *et al* which included 240,203 subjects (24 studies) who received BCG-vaccination during infancy reported that only 1% was TST-positive 10 years after BCG vaccination, suggesting that its effect is insignificant [13]. Since our subjects were at the age of 11 or older, and most of them received BCG vaccination during infancy, TST -positive rates were significantly higher than IGRA-positive rates. However, it is difficult to conclude that there are high TST false-positive rates given the fact that when the TST at 15 mm cutoff was used, it yielded a higher odds ratio.

The incidences of tuberculosis based on initial QFT and TST-positive statuses were not significantly different between subjects diagnosed in the first year of enrolment and those afterwards. This may be due to the fact that follow-ups were not long enough to detect a drop in risk.

Of 215 subjects with tuberculosis who received chemotherapy at the inception, 5 progressed to active tuberculosis and 4 had a low patient adherence (Figure 1). Three of the 5 students who initiated preventive treatment showed TST+, QTF-GIT + results at the first enrollment; however, they received preventive treatment to achieve optimal care. These occurrences are preventive chemical treatment, respectively, one month, three months after his aborted because it was caused by chemical treatment and prevention. Only 1 student in the cohort group achieved optimal care. Low compliance with medical treatment of tuberculosis is a factor for treatment failure and an increase in new tuberculosis cases. INH therapy had been performed to prevent tuberculosis by many researchers in the mid-1950s but the effect of this strategy is beyond the scope of this study.

Diel [14] demonstrated that commercial IGRAs have higher positive and negative predictive values for progression to active tuberculosis compared with those of the TST, especially when performed in high-risk persons. However, the results of our study are not consistent with this. This may be attributed to the difference in the subjects included.

In multivariate analysis of risk factors associated with the TST- and GFT-GIT-positive rate, none of the risk factors (including type of contact and the BMI) were statistically significant. Also, data analysis showed that positive responders to both TST and QFT-GIT had 7.96 times (95% CI; 3.14-20.22) more risk for the development of tuberculosis, which demonstrates that this group should be subjected to preventive treatment as in the current national guideline.

## Conclusions

Results of this study likewise support the current recommendation other study of using the TST 15 mm cutoff for patients aged 11–17 years. The OR of QFT-GIT for the development of active tuberculosis and its intermediate agreement with TST using 15 mm cutoff demonstrates its role as an adjunct diagnostic tool to current clinical practice. Positive responders to both TST and QFT-GIT at the outset may benefit from chemoprophylaxis. More studies are needed to demonstrate the promising role of QFT-GIT in the Korean setting.
